# Bletilla Striata Polysaccharide Alleviates Neutropenia by Promoting C/EBPε-Dependent Hematopoietic Reconstitution

**DOI:** 10.3390/cells14231888

**Published:** 2025-11-28

**Authors:** Yaru Cui, Yingying Luo, Cheng Zhang, Dan Shan, Yulin Feng, Shilin Yang, Lanying Chen, Jun Yu

**Affiliations:** 1 National Pharmaceutical Engineering Center for Solid Preparation in Chinese Herbal Medicine, Jiangxi University of Chinese Medicine, Nanchang 330006, China; cuiyaru@jxutcm.edu.cn (Y.C.);; 2 Jiangxi Provincial Key Laboratory of Effective Material Basis of TCM, Jiangxi University of Chinese Medicine, Nanchang 330004, China; 3 Department of Cardiovascular Sciences and Center for Metabolic Disease Research, Lewis Katz School of Medicine, Temple University, Philadelphia, PA 19140, USA

**Keywords:** neutropenia, BSP, HSPCs, C/EBPε, hematopoietic reconstitution

## Abstract

Neutropenia is a common complication in oncology patients receiving chemotherapy, and rapid regeneration of functional neutrophils is critical for effective management. Bletilla striata polysaccharide (BSP) has shown therapeutic potential, but its mechanisms and molecular targets remain unclear. Here, we demonstrate that BSP accelerates the recovery of white blood cells, particularly neutrophils, in a chemotherapy-induced neutropenia (CIN) mouse model with cyclophosphamide (CY). The regenerated neutrophils retained phagocytic activity against bacteria, and BSP treatment significantly reduced mortality in the endotoxin-induced mouse death model. Furthermore, BSP enhanced the repopulation of hematopoietic stem and progenitor cells (HSPCs) in the bone marrow and promoted cell-cycle entry, resulting in increased frequencies of long-term hematopoietic stem cells (LT-HSCs), multipotent progenitors 2 (MPP2), and MPP3/4 subsets. Both in vitro colony formation and in vivo competitive transplantation assays confirmed that BSP reshapes hematopoietic reconstitution and corrects aberrant myeloid differentiation. PCR array analysis of HSPCs indicated that this process is mediated by C/EBPε and its downstream genes (LTF, LCN2, and ELANE). Consistently, BSP failed to support myeloid reconstitution following C/EBPε knockdown in vitro. In a C/EBPε knockout mouse model, HSPCs repopulation and regeneration were impaired, and BSP failed to promote neutrophil recovery after CY challenge or the mobilization of MPP2 and MPP3/4 subsets. The regulatory effects of BSP on C/EBPε target genes were also abolished. In conclusion, our findings identify C/EBPε as a key mediator of BSP activity, driving HSPCs repopulation and restoring hematopoietic function. These results highlight BSP as a potential therapeutic strategy for chemotherapy-induced neutropenia.

## 1. Introduction

Neutropenia, the most common complication of chemotherapy in oncology patients, is referred to as chemotherapy-induced neutropenia (CIN) [1]. It is characterized by a marked reduction in circulating neutrophils, the primary cellular mediators of innate immunity [2,3]. Consequently, neutropenia remains a major clinical challenge, as it is complicated by substantial morbidity and mortality. Effective therapeutic approaches that can induce sufficient numbers of functional neutrophils without increasing the risk of myeloid malignancy are urgently needed.

One promising strategy against CIN is to modulate the differentiation of hematopoietic stem cells (HSCs) toward neutrophil production. HSCs, residing primarily in the bone marrow niche, are the only cells capable of generating all blood lineages throughout life. Within this microenvironment, HSCs interact with endothelial cells, mesenchymal stromal cells (MSCs), and other niche components [4]. Hematopoietic differentiation proceeds from primitive long-term HSCs (LT-HSCs) through intermediate progenitors, including multipotent progenitors (MPP2 and MPP3/4), ultimately giving rise to lineage-committed progenitors and mature blood cells [5]. Under steady-state conditions, HSCs are largely quiescent [6]. However, in response to stressors, such as chemotherapy or radiotherapy, they are rapidly activated by regulatory signals within the niche to replenish hematopoietic cells.

Granulocyte colony-stimulating factor (G-CSF) is the most widely used cytokine and has been a mainstay of CIN management for over two decades by shortening the duration of neutropenia [7,8,9]. However, G-CSF therapy has notable drawbacks, including bone pain, flushing, and nausea. Importantly, G-CSF-expanded neutrophils often display impaired differentiation and defective microbicidal function [10,11,12]. Recent studies suggest that polysaccharides derived from natural plants possess hematopoietic activity [13,14]. Bletilla striata polysaccharide (BSP) is a major bioactive component of the traditional Chinese medicinal herb Bletilla striata. According to the Chinese pharmacopeia, Bletilla striata is documented to have clinical applications such as astringing, stopping bleeding, reducing swelling, and promoting tissue regeneration. Pharmacological studies have also demonstrated its anti-inflammatory and immune-enhancing effects [15,16,17]. Despite these observations, whether BSP exerts hematopoietic effects, particularly in the context of CIN, remains unclear. Here, we investigated the role of BSP in promoting HSPCs reconstitution and its potential to restore neutrophil production in chemotherapy-induced neutropenia.

## 2. Materials and Methods

### 2.1. Cell Lines, Drugs, and Antibodies

293T and 3T3 cell lines were obtained from the American Type Culture Collection (ATCC, Manassas, VA, USA). Cells were maintained in DMEM (Gibco, Waltham, MA, USA), which was supplemented with 10% fetal bovine serum (Gibco, Waltham, MA, USA). All cells were cultured at 37 °C in a humidified atmosphere containing 5% CO_2_.

BSP was obtained from Jiangxi University of Chinese Medicine (Nanchang, China). The total carbohydrate content of BSP was ≥ 98%, determined by phenol–sulfuric acid colorimetry. High-performance gel permeation chromatography (HPGPC) analysis showed a single symmetrical peak, confirming homogeneity, with an estimated molecular weight of 48.3 kDa (Shimadzu LabSolutions software, version 6.83, Kyoto, Japan). Gas chromatography–mass spectrometry (GC-MS) identified mannose and glucose as the monosaccharide components at a 2:1 molar ratio.

rhG-CSF (Amoytop biotech, Xiamen, China) was used. Flow cytometer antibodies against Lineage (#22-7770-72), c-Kit (#171171), Sca-1 (#125981), CD48 (#563536), and CD150 (#25-1502) were purchased from eBioscience Co., Ltd. (SanDiego, CA, USA). Anti-CD45.1 (#E-AB-F1184E), CD45.2 (#E-AB-F1122H), CD3 (#E-AB-F1013Q), CD45R/B220 (#E-AB-F1112D) were obtained from Elabscience (Wuhan, China) and CD11B (BD Biosciences, Franklin Lakes, NJ, USA, #55736). Ki67 antibody (#561277) and HSP90 (#610419) were purchased from BD Co., Ltd. (Franklin, NJ, USA). C/EBPε antibody (#sc-515192) was purchased from Santa Cruz Co., Ltd. (Santa Cruz, CA, USA).

### 2.2. Animal Experiments

Under the guidelines of the protocol approved by CHLA Institutional Animal Care, SPF C57BL/6J male mice (18–20 g, 6–8 weeks old) were purchased from Jackson Laboratory and housed in a controlled, air-conditioned environment with a 12 h light–dark schedule. Animals were provided with access to sufficient food and water.

Cyclophosphamide-induced neutropenia mouse model: Neutropenia was induced in mice with a single intraperitoneal injection of cyclophosphamide (CY; Sigma, St. Louis, MO, USA, #AC203960000) at 150 mg/kg on day 1, as previously described [18,19]. Control mice received an equivalent volume of saline. From day 2 onward, mice were treated once daily with one of the following regimens: recombinant human G-CSF (rhG-CSF; 20 μg/kg, subcutaneous), BSP (30 or 60 mg/kg, intravenous; CY + BSP groups), or saline (CY group). BSP was dissolved in saline at different concentrations and then was filtered through a 0.22 μm filter before injection. Treatments continued for 2 or 5 consecutive days, depending on the experimental design. Peripheral blood (30 μL) was collected every 2 days via retro-orbital venous plexus sampling under anesthesia and analyzed using an automated hematology analyzer (HEMAVET 950FS, Drew Scientific, Oxford, CT, USA).

LPS challenge experiment: Neutropenia was induced in mice with a single intraperitoneal injection of CY on day 1, firstly. Five hours later, mice received BSP (60 mg/kg) or saline, as described above. After 3 h, both neutropenic and normal mice were injected intraperitoneally with lipopolysaccharide (LPS, 22.5 mg/kg in saline, Sigma, St. Louis, MO, USA, #L2630) [20]. BSP treatment was repeated for 4 days, and survival was recorded once daily.

Competitive bone marrow transplantation: Bone marrow injury was induced in 8-week-old male CD45.1 mice (purchased from Ziyuan Experimental Animal Technology, Hangzhou, China) with cyclophosphamide (300 mg/kg) [21,22]. The mice were then administered with either saline or BSP (60 mg/kg) for 5 consecutive days. On day 21, the bone marrow cells from the above-treated mice were mixed with those from normal CD45.2 mice at a 1:1 ratio. A total of 2 × 10^6^ cells were transplanted into lethally irradiated (7 Gy) CD45.2 recipient mice via tail vein injection. Donor chimerism and lineage distribution in peripheral blood were evaluated every 4 weeks by flow cytometry.

C/EBPε knockout mice: C/EBPε-deficient mice on a C57BL/6J background were generated using CRISPR/Cas9 (Cyagen Biosciences, Guangzhou, China). CY and BSP (60 mg/kg) treatments were performed as described above.

### 2.3. Neutrophils Isolation and Giemsa Staining

Mice were euthanized after BSP treatment (60 mg/kg) for 5 days. A total of 100 μL of whole blood was collected and transferred to a 1.5 mL tube containing 1 mmol/L sodium pyruvate. Neutrophils were harvested by Ficoll (GE, Fairfield, CT, USA, #17544602) and smears were prepared by the routine method. Fix smears in absolute methanol for 3 min at room temperature, stain with Giemsa (Solarbio, Beijing, China, #G1010) for 15 min, rinse smears with running water, air-dry, and seal with neutral balsam (Solarbio, Beijing, China, #G8590). Images were captured by an optical microscope (Leica, Wetzlar, Hesse, Germany).

### 2.4. Phagocytosis Assay

Mice were treated using the same method as above. A total of 50 μL of whole blood was collected and transferred to a 1.5 mL tube containing 1 mmol/L sodium pyruvate on day 7. Lysis of erythrocytes was performed using ACK lysing buffer (ThermoFisher, Waltham, MA, USA, #00-4300-54), and samples were then incubated with pHrodo FITC-labeled S. aureus (ThermoFisher, Waltham, MA, USA, #P35367) at 37 °C or 4 °C for 1 h. After incubation, the percentage of bacteria-ingesting cells and the median fluorescence intensity (MFI) of phagocytosis were calculated using a BD Caliber flow cytometer (BD Biosciences, Franklin Lakes, NJ, USA). MFI is calculated as the sum of fluorescence intensity values from all analyzed cells divided by the total number of cells. This metric serves to normalize bacterial uptake to the cell number, providing a quantitative measure of phagocytic activity.

### 2.5. Flow Cytometry Analysis

On day 3, prior to neutrophil recovery, for the determination of HSPCs frequency in the bone marrow, mice were euthanized after BSP treatment (60 mg/kg) twice. Then, bone marrow cells were isolated from the femora, tibiae, and ilia by gently crushing in PBS with 2% FBS using a syringe connected with the 25 G needle on day 3. The cells were processed with 40 μm filter (BD Biosciences, Franklin Lakes, NJ, USA), followed by using ACK lysing buffer to remove erythrocytes. After that, samples were washed twice in PBS and incubated for 30 min on ice using the following surface antibodies: anti-Lineage, anti-c-Kit, anti-Sca-1, anti-CD48, and anti-CD150. The cells were defined as LSKs (Lin-Kit + Sca-1+), LT-HSCs (LSK CD48-CD150+), MPP2 (LSK CD48 + CD150+), and MPP3/4 (LSK CD48 + CD150-).

For cell-cycle analysis, bone marrow cells were harvested using the aforementioned method and stained with surface marker antibodies. Subsequently, fixation and permeabilization were performed using the Transcription Factor Staining Buffer Set (eBioscience, SanDiego, CA, USA, #00-5523). Nuclear staining of Ki67 was performed using Ki67 antibody. Finally, the cells were stained with Hoechst 33342 (ThermoFisher, Waltham, MA, #USAR37165) for 5 min, which were then analyzed using an LSR II (BD Biosciences, Franklin Lakes, NJ, USA) or sorted on an Aria II (BD Biosciences, Franklin Lakes, NJ, USA).

In competitive transplantation experiments, 30 μL of whole blood was collected in the 4th and 8th weeks after transplantation. Lysis of erythrocytes was performed using ACK lysing buffer, and samples were then incubated with anti-CD45.1, CD45.2, CD3, CD45R/B220, and CD11B at 4 °C for 30 min. After incubation, the percentage of cells was calculated using a flow cytometer (Gallios, Beckman, Brea, CA, USA).

### 2.6. Live Imaging

Initially, on day 3, LSK cells from different treatment group mice were sorted and seeded on 8-well chamber slide (ibidi, Martinsried, Germany). The environmental chamber was maintained at 37 °C, 5% CO_2_. Using the Leica TCS SP8 system (Leica, Wetzlar, Hesse, Germany), LSK cell division was tracked across 15 non-overlapping wide fields for 48 h with a 20× objective. Cell division was recorded as a video and analyzed the using Fiji software (version 2.0.0, Madison, WI, USA).

### 2.7. Colony-Forming Unit (CFU) Assay

On day 3, mouse bone marrow cells from different treatment mice were isolated and lysed by ACK buffer as described above. 2000 BM cells were cultured in a 3.5 cm dish with 3 mL Metho Cult M3434 medium (Stem Cell Technologies, Vancouver, BC, Canada, #03434). After BM cells were plated for 8 days, colony formation was observed and quantified with a microscope (Leica, Wetzlar, Hesse, Germany, DMi 1).

### 2.8. PCR Array Analysis and Quantitative Real-Time PCR

HSPCs (LSK CD150+) were sorted on day 3 following BSP treatment (60 mg/kg) and total RNA was isolated using the RNeasy Plus Mini Kit (Qiagen, Hilden, Germany, #74034). A mouse hematopoiesis PCR array (Qiagen, Hilden, Germany, # PAMM-054Z) was performed according to the manufacturer’s instructions.

For the quantitative real-time PCR test, Lineage-negative cells were enriched by magnetic beads (Miltenyi Biotec, Bergisch Gladbach, NRW, Germany, #130-110-470) from the bone marrow of differentially treated mice on day 3. Total RNA of lineage-negative cells from experiment mice was isolated first, and cDNA was transcribed with reverse transcriptase (ThermoFisher, Waltham, MA, USA, #18080-051). Real-time PCR was performed using the SYBR Green Mix (Bio-Rad, Hercules, CA, USA) on a CFX96 quantitative PCR instrument (Bio-Rad, Hercules, CA, USA, CFX96). Relative mRNA expression was normalized to β-actin. Primers for real-time PCR are listed in Table S1.

### 2.9. C/EBPε shRNA Construction and Transfection

To investigate the function of C/EBPε in the HSPCs. LSK cells were transfected with shRNA-targeting C/EBPε to silence C/EBPε. C/EBPε-specific targeting shRNAs were synthesized and (refer to Table S1 for sequences) constructed into a retroviral vector (PGLV3-H1-GFP). Recombinant lentiviral particles were produced in 293T cells by transfecting PGLV3-H1-GFP plasmid containing the C/EBPε shRNA sequence, as well as helper plasmids including PEMV-VSVG and pCMV-8.91. The virus medium was harvested after 48 h of transfection, filtered through a 0.45 μm filter, and then concentrated using the Lenti-X Concentrator (TaKaRa, Kyoto, Japan, #631231).

LSK cells from WT C57BL/6J mice were sorted using Aria II before adding the concentrated C/EBPε shRNA virus medium with 8 μg/mL polybrene (Sigma, St. Louis, MO, USA, #H9268) in complete stem cell medium (low glucose DMEM, containing 100 ng/mL SCF, 100 ng/mL TPO, and 100 ng/mL Flt3-Ligand). SCF (#250-03), TPO (#315-14), and Flt3-Ligand (#P250-32L) were purchased from PEPROTECH Co., Ltd. (Cranbury, NJ, USA). After 72 h infection, LSK cells were harvested and plated with a density of 300 cells cultured in Metho Cult M3434 medium with PBS or BSP (20 μg/mL). Colony formation was scanned and quantified using Confocal 8 days after the initial inoculation.

### 2.10. Western Blotting

To detect the efficiency of C/EBPε shRNA, 3T3 cells were infected 72 h after being treated with the virus medium containing C/EBPε shRNA. Total protein was collected before being fractionated on SDS-PAGE and transferred to a nitrocellulose membrane by electroblotting. The membrane was blocked with 5% BSA (Sigma, St. Louis, MO, USA, #A7030) in TBST for 1 h, followed by overnight incubation at 4 °C with a primary antibody against C/EBPε. HSP90 was used as an internal control.

### 2.11. Statistical Analysis

Statistical analysis was performed by one-way ANOVA for comparisons with more than two groups, or unpaired Student’s *t*-test (two tails) for comparisons between two groups. Prior to analysis, the assumptions of normality and homogeneity of variances were verified. Normality was assessed using the Shapiro–Wilk test, and homogeneity of variances was confirmed using Levene’s Test. For data that passed both the normality and variance tests, unpaired Student’s *t*-test or one-way ANOVA was used, followed by Bonferroni’s post hoc test. For data that passed the normality test but failed the variance test, Brown–Forsythe and Welch’s ANOVA tests were used, followed by Dunnett’s T3 multiple comparison test. For data that failed the normality test, Mann–Whitney U test was used. Graph Pad Prism 9 (Version 9.0; La Jolla, CA, USA) was used for statistical analysis. *p* < 0.05 was considered a statistically significant difference. The sample size was estimated using the G*Power software (version 3.1.9.6, Düsseldorf, Germany), employing an effect size derived from preliminary experiments or the literature, using a significance level of 0.05 and a power of 0.8. All experiments were performed at least in triplicates.

## 3. Results

### 3.1. BSP Accelerates Recovery of Peripheral Blood Cells by Promoting Functional Neutrophil Production in Neutropenia Mice

**Figure 1:**
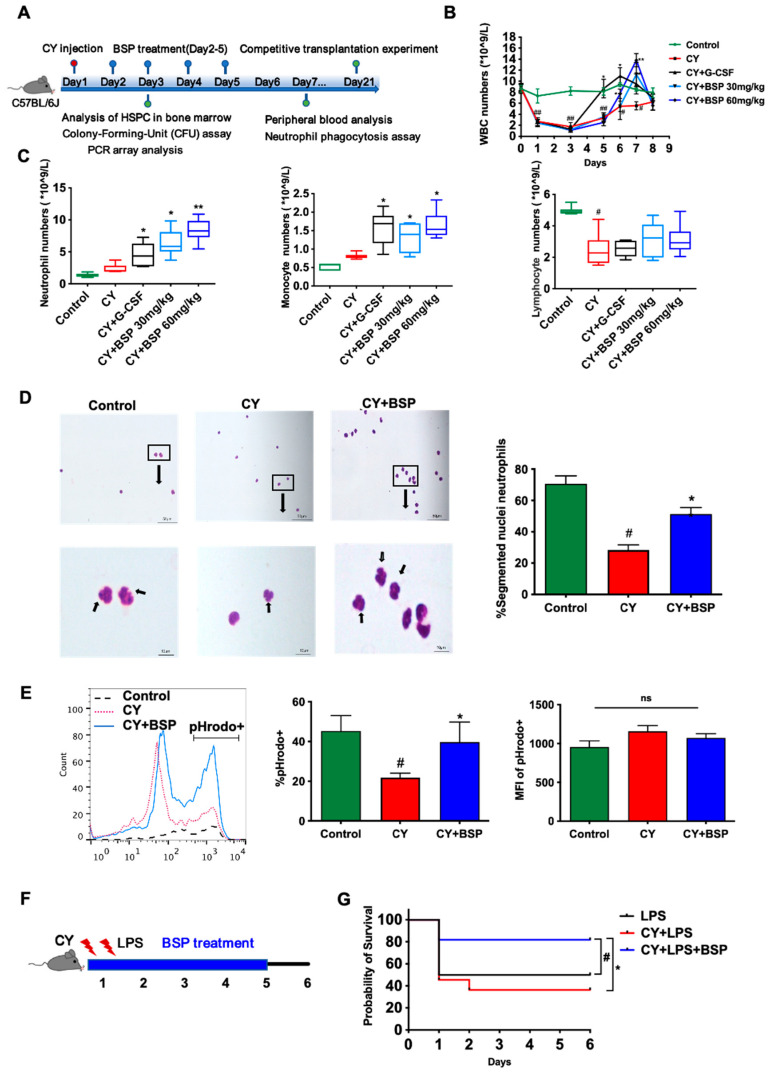
Bletilla striata polysaccharide (BSP) accelerates the recovery of peripheral blood cells in neutropenia mice by stimulating functional neutrophil production. (**A**) Experimental timeline of this study. (**B**,**C**) BSP increased white blood cell (WBC) numbers in a dose-dependent manner in chemotherapy-induced neutropenia (CIN) mice. Mice were injected with the chemotherapy drug cyclophosphamide (CY) to induce CIN. Regimen treatments were applied from day 2 to day 5, and peripheral blood was collected and analyzed every two days. Data are presented as median (±interquartile range). Statistical significance was determined by Mann–Whitney U test (**C**); # *p* < 0.05 versus control group; * *p* < 0.05 or ** *p* < 0.01 versus CY group. (**D**) Neutrophils harvested with ficoll from PB were stained with Giemsa on day 7. According to the morphological characterization, the percentage of segmented nuclei was counted. (**E**) PB was lysed by ACK lysis buffer and incubated with pHrodo FITC-labeled S. aureus at 37 °C for 1 h. The percentages and MFI of pHrodo+ neutrophils were calculated by flow cytometer. Data are expressed as the mean ± SD, *n* = 3; statistical significance was determined by one-way ANOVA with Bonferroni’s post hoc test (**B**,**D**,**E**); # *p* < 0.05 or ## *p* < 0.01 versus control group; * *p* < 0.05 or ** *p* < 0.01 versus CY group; ns, *p* > 0.05. (**F**) Experimental timeline of survival study. (**G**) BSP ameliorates endotoxin-induced mouse death. After an initial injection of CY to induce neutropenia, mice were treated with either BSP (60 mg/kg) or saline five hours later; all animals were then subjected to an LPS challenge three hours after that to assess survival rate. Survival analysis was performed using the Kaplan–Meier method, and the log-rank test was used for comparison. LPS group mice *n* = 6; CY/CY + BSP group mice *n* = 10; # *p* < 0.05 versus LPS group; * *p* < 0.05 versus CY + LPS group.

To investigate whether BSP plays a role in regulating hematopoiesis to resist neutropenia, we first established a cyclophosphamide-induced neutropenia mouse model. As expected, the decline phase (days 1–3) was characterized by a decrease in white blood cell counts, which then gradually recovered during the recovery phase (days 4–7) (Figure 1B). BSP administration significantly accelerated white blood cell recovery in a dose-dependent manner, at 30 or 60 mg/kg. Therefore, a dosage of 60 mg/kg was consistently employed in all subsequent studies. On day 7, the most pronounced effect of BSP was observed in neutrophil regeneration following CY-induced depletion, comparable to G-CSF. Monocyte counts increased modestly, while lymphocyte counts remained unchanged (Figure 1C).

In this model, a significant number of neutrophils may also be generated as a result of the organism’s self-stress response. To assess the functionality of regenerated neutrophils, we performed Giemsa staining and in vitro phagocytosis assays. Morphologically, neutrophils from CY-treated mice displayed incomplete terminal characterized by large or indented nuclei. In contrast, BSP-treated or control mice exhibited more mature neutrophils with segmented nuclei (Figure 1D). In the phagocytosis assay, the proportion of pHrodo^+^ bacteria-ingesting neutrophils was significantly higher in BSP-treated mice. However, the median fluorescence intensity (MFI), reflecting phagocytic activity, did not differ from CY-treated controls (Figure 1E). These findings indicate that BSP promotes the generation of functional, phagocytically competent neutrophils, without enhancing phagocytic strength.

Finally, we evaluated whether BSP could reduce endotoxin-induced mouse death. In an endotoxin challenge, BSP treatment markedly improved survival of CIN mice, with approximately 80% survival compared to about 30% in the CY + LPS group (Figure 1G). These results demonstrate that BSP not only accelerates neutrophil recovery but also confers protection against infection during chemotherapy-induced neutropenia.

### 3.2. BSP Promotes the Expansion of the Hematopoietic Stem and Progenitor Cells Pool in the Bone Marrow

**Figure 2:**
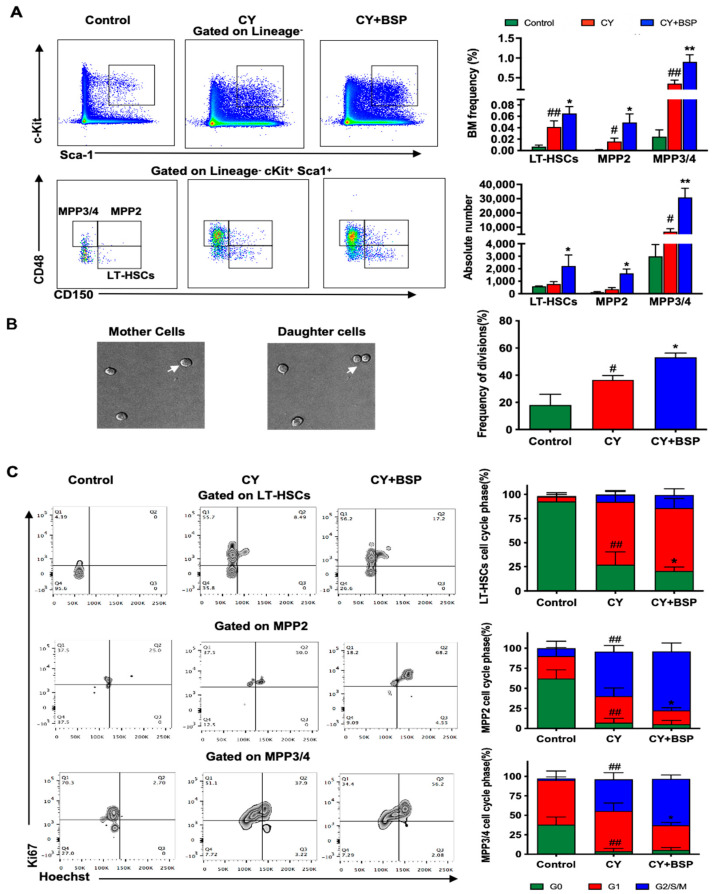
BSP promotes the expansion of the hematopoietic stem and progenitor cells (HSPCs) pool in the bone marrow. (**A**) Bone marrow cells were harvested on day 3, subjected to RBC lysis, stained with surface marker-specific antibodies for 30 min on ice, and subsequently analyzed via flow cytometry to determine the frequencies of distinct HSPCs subsets. (**B**) BSP enhances HSPCs division and activation. On day 3, LSK cells from mice in different treatment groups were sorted and seeded on an 8-well chamber slide, which was maintained at 37 °C and 5% CO_2_ for tracking LSK cells division in 15 non-overlapping wide fields for 48 h with the Leica TCS SP8 system. See also the Supplementary Video. (**C**) On day 3, bone marrow cells from each group were first stained with surface marker antibodies, then fixed and permeabilized for staining with Ki67 and Hoechst 33342 to analyze the cell cycle of distinct HSPCs subsets by flow cytometry. Data are expressed as mean ± SD, *n* = 3; statistical significance was determined by one-way ANOVA with Bonferroni’s post hoc test (**A**–**C**); # *p* < 0.05 or ## *p* < 0.01 versus control group; * *p* < 0.05 or ** *p* < 0.01 versus CY group.

HSPCs either self-renew or give rise to multipotent cells whose progeny provides precursors committing to the lymphoid and myeloid lineages. To test whether BSP induces HSPCs expansion, on day 3, prior to neutrophil recovery, we quantified the proportion of HSPCs in the bone marrow to assess their potential contribution to the neutrophil population observed on day 7. We found that both the frequency and absolute numbers of LT-HSCs, MPP2, and MPP3/4 had considerable elevations (Figure 2A). Although CY injection was an inducer of active HSPCs, BSP promoted this activation to a greater extent, inducing HSPCs proliferation and differentiation.

Similar findings were observed when we screened the division of HSPCs ex vivo in real-time using live imaging, showing that LSK cells from bone marrow in BSP-treated mice had a higher division frequency compared with CY group mice (Figure 2B). In addition, to characterize the progression of HSPCs, we examined the activation of the cell cycle machinery in LT-HSCs, MPP2, and MPP3/4 through Ki67 and Hoechst 33342 staining. Consistent with this, we found a reduction in G0 phase ratio in LT-HSCs and an increase in the G2/S/M phases in MPP2 and MPP3/4 cells, indicating BSP accelerated LT-HSCs entry into the cell cycle for expansion and further induced MPP2 and MPP3/4 division (Figure 2C).

### 3.3. BSP Enhances the Hematopoietic Functional Capacity of HSPCs

**Figure 3:**
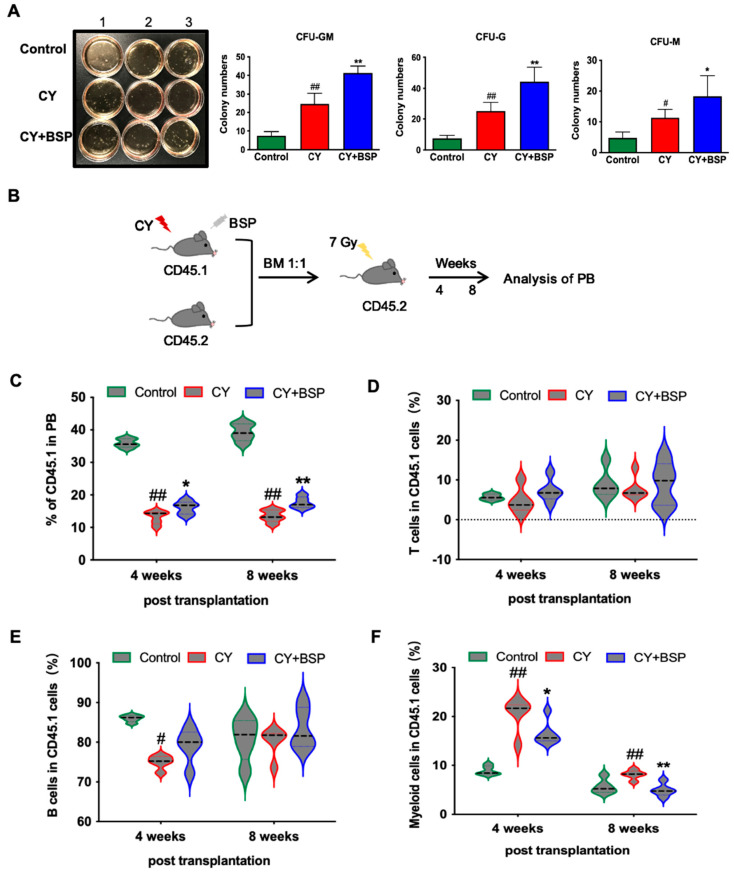
BSP enhances the hematopoietic functional capacity of HSPCs. (**A**) On day 3, bone marrow cells were harvested from mice and plated in the MethoCult™ M3434 medium. After 8 days of culture, colony numbers were quantified under the microscope. Data are expressed as the mean ± SD, *n* = 3; statistical significance was determined by one-way ANOVA with Bonferroni’s post hoc test (**A**); # *p* < 0.05 or ## *p* < 0.01 versus control group; * *p* < 0.05 or ** *p* < 0.01 versus CY group. (**B**) Experimental design of competitive transplantation. (**C**) The donor population was analyzed in the peripheral blood. (**D**–**F**) Donor-derived lineage chimerism in PB from transplanted recipients was analyzed, including T lymphocytes (**D**), B lymphocytes (**E**), and myeloid cells (**F**). Data distribution is shown as violin plots, *n* = 5; statistical significance was determined by one-way ANOVA with Bonferroni’s post hoc test (**C**–**F**); # *p* < 0.05 or ## *p* < 0.01 versus control group; * *p* < 0.05 or ** *p* < 0.01 versus CY group.

To determine whether the HSPCs expanded by BSP treatment possess functional hematopoietic reconstitution capacity, we performed complementary in vitro clonal assays (CFU assays) and in vivo competitive bone marrow transplantation experiments. CFU analysis revealed that BSP increased total colony numbers, specifically granulocyte/macrophage (GM)-type colonies and granulocyte (G)-type colony numbers (Figure 3A), indicating HSPCs from BSP-treated mice are prone to myeloid differentiation compared with the CY group.

To assess the functional capacity of HSPCs following BSP treatment in vivo, we conducted a competitive bone marrow transplantation assay. Donor bone marrow cells from CD45.1 mice, which were treated with BSP or saline and subjected to a severe CY injury, were mixed with competitor cells from wild-type (WT) CD45.2+ mice and transplanted into lethally irradiated CD45.2 recipient mice (Figure 3B). The lineage composition (myeloid, B, and T cells) of donor-derived (CD45.1+) cells was analyzed in the peripheral blood of recipients (Figure 3C–F). After high-dose CY-induced bone marrow injury, the chimerism of CD45.1+ donor cells was significantly lower than that of controls at 4 and 8 weeks post-transplantation (Figure 3C), indicating that CY treatment impaired the reconstitution capacity of HSPCs. Furthermore, CY injury resulted in aberrant lineage output, characterized by a significant reduction in the proportion of B cells within the CD45.1+ cells at week 4 (Figure 3E). In contrast, the proportion of T cells remained unchanged (Figure 3D). Notably, treatment with BSP significantly restored normal myeloid differentiation and the overall hematopoietic reconstitution capacity of the donor HSPCs (Figure 3F).

### 3.4. BSP Upregulates C/EBPε Expression in HSPCs

**Figure 4:**
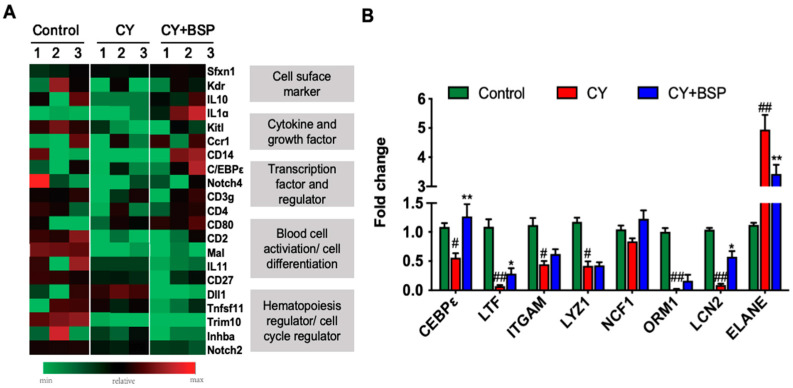
BSP upregulates C/EBPε expression of HSPCs. (**A**) PCR array data analysis of HSPCs. HSPCs were sorted on day 3 and subjected to RNA isolation, and a mouse hematopoiesis PCR array was performed. (**B**) BSP significantly upregulates the expression of C/EBPε, along with its regulated downstream genes lactotransferrin(LTF), lipocalin-2 (LCN2), and downregulated neutrophil elastase (ELANE) in lineage-negative cells. Lineage-negative cells were enriched by magnetic beads from the bone marrow of differentially treated mice on day 3. Real-time PCR was performed to determine the expression levels of the relevant genes. Data are expressed as the mean ± SD, *n* = 4; statistical significance was determined by Brown–Forsythe and Welch’s ANOVA tests with Dunnett’s T3 multiple comparison test; # *p* < 0.05 or ## *p* < 0.01 versus control group; * *p* < 0.05 or ** *p* < 0.01 versus CY group.

To elucidate the molecular mechanism by which BSP promotes hematopoietic reconstitution by regulating hematopoietic stem and progenitor cells, we analyzed the gene expression profile of HSPCs using a mouse hematopoiesis pathway PCR array. Out of 84 genes, 37 were differentially expressed (> 1.2-fold change) in BSP-treated cells. Among these, 21 genes showed either a > 1.5-fold change or a > 1.2-fold change with statistical significance, including thirteen upregulated and eight downregulated genes (Figure 4A; Table S2).

HSPCs fate is tightly regulated by intrinsic transcription factors and signaling pathways. Among the differentially expressed genes, C/EBPε emerged as a critical transcription factor. It is well established that C/EBPε is a key regulator of myeloid differentiation and neutrophil function [23,24]. Previous studies demonstrated that C/EBPε expression in human CD34^+^ hematopoietic stem cells drives granulocytic differentiation under appropriate cytokine stimulation and that acetylation of C/EBPε in CD34^+^ progenitors influences lineage commitment toward the myeloid lineage [25,26]. Consistent with this, our analysis of lineage-negative cells showed that BSP treatment significantly upregulated C/EBPε expression, as well as its downstream targets of lactotransferrin (LTF) and lipocalin-2 (LCN2) by a four-fold and a six-fold increase, respectively. It also downregulated neutrophil elastase (ELANE) expression by 1.5-fold (Figure 4B). These results suggest that BSP promotes neutrophil differentiation and function, at least in part, through the induction of C/EBPε and its transcriptional program.

### 3.5. BSP-Enhanced HSPCs Colony Formation In Vitro Is C/EBPε-Dependent

**Figure 5:**
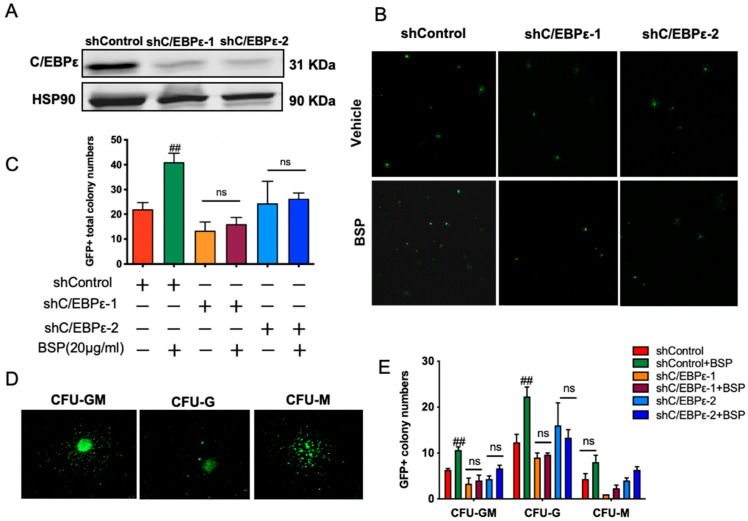
C/EBPε knockdown abrogated the ability of BSP to promote the colony formation of HSPCs in vitro. (**A**) The expression levels of C/EBPε protein were detected by Western blotting after shRNA transfection for 72 h in 3T3 cells. (**B**) LSK cells from wild-type C57BL/6J mice were sorted and then transduced with concentrated C/EBPε shRNA viral preparation in complete stem cell medium. After 72 h of infection, the transduced LSK cells were harvested and plated in MethoCult™ M3434 medium at a density of 300 cells per dish with either PBS or BSP (20 μg/mL). After 8 days of culture, GFP-positive colonies were observed and quantified using confocal scanning. (**C**) Frequency of GFP-positive total colony numbers in different treatment cells. (**D**) Representative morphology of different colony types observed. (**E**) Frequency of GFP-positive colonies of different clonal types. Data are expressed as the mean ± SD, *n* = 3. Statistical significance was determined by one-way ANOVA with Bonferroni’s post hoc test (**C**, **E**); ## *p* < 0.01 versus control group; ns, *p* > 0.05.

Building on the above findings, we reasoned that BSP promotes HSPCs expansion and differentiation to strengthen hematopoietic function during neutropenia, and that this effect may depend on C/EBPε. To test this, LSK cells were transduced with lentivirus-mediated shRNAs targeting C/EBPε. To confirm the efficacy of shRNAs, we validated the shRNA-mediated knockdown of C/EBPε mRNA in LSK cells (Figure S5B). Additionally, we assessed the functional effects of these shRNAs in 3T3 cells. Two independent shRNA constructs (shRNA1 and shRNA2) markedly reduced C/EBPε expression at 72 h post-transfection (Figure 5A).

Colony-forming assays were then performed to evaluate the role of C/EBPε in BSP-mediated hematopoietic activity. BSP (20 μg/mL) significantly increased the number of colonies compared with untreated controls (Figure 5B,C), particularly enriching GM- and G-type colonies (Figure 5D,E). In contrast, C/EBPε knockdown completely abolished this effect, showing that BSP failed to enhance hematopoietic reconstitution under these conditions. These findings demonstrate that the ability of BSP to stimulate myeloid expansion and colony formation is critically dependent on C/EBPε, highlighting its essential role in mediating BSP-driven neutrophil regeneration.

### 3.6. C/EBPε Is Essential for BSP-Mediated Hematopoietic Reconstitution In Vivo

C/EBPε plays a critical role in myeloid development, particularly during the transition from promyelocytes to myelocytes [27,28]. C/EBPε knockout (C/EBPε^−/−^) mice exhibit impaired neutrophil migration across the peritoneal membrane [29,30] and are more susceptible to bacterial infections, often resulting in reduced survival [31]. Nevertheless, its regulatory functions in bone marrow hematopoiesis remain poorly understood.

**Figure 6:**
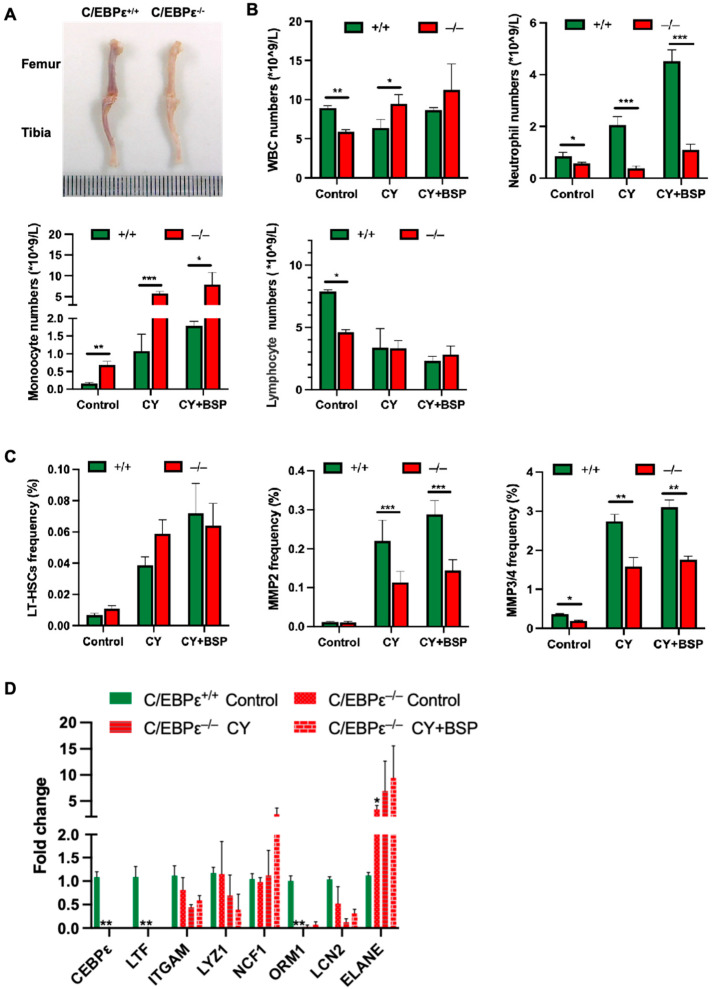
BSP attenuates neutrophil recovery capacity and expansion of HSPCs after depletion of C/EBPε. (**A**) Femurs and tibias of wild-type mice (C/EBPε^+/+^) and C/EBPε knockout mice (C/EBPε^−/−^). (**B**) C/EBPε^−/−^ mice were injected with 150mg/kg CY to induce CIN. From day 2 until day 5, 60 mg/kg BSP treatment was also performed, and peripheral blood was collected and analyzed on day 7. (**C**) The frequency of LT-HSCs, MPP2, and MPP3/4 cells in the bone marrow of C/EBPε^−/−^ mice was analyzed by flow cytometry on day 3. Data are expressed as the mean ± SD, *n* = 3; statistical significance was determined by one-way ANOVA with Bonferroni’s post hoc test (**B**,**C**); * *p* < 0.05, ** *p* < 0.01, and *** *p* < 0.001 versus C/EBPε^+/+^ group. (**D**) Lineage-negative cells were enriched by magnetic beads from the bone marrow of differentially treated C/EBPε^−/−^ mice on day 3. Real-time PCR was performed to quantify the expression levels of CEBPε and the down-regulated target genes. Data are expressed as the mean ± SD, *n* = 3; statistical significance was determined by Brown–Forsythe and Welch’s ANOVA tests with Dunnett’s T3 multiple comparison test; * *p* < 0.05 or ** *p* < 0.01 versus C/EBPε^+/+^ control group.

To further explore whether BSP ameliorates HSPCs dysfunction via C/EBPε, we generated C/EBPε^−/−^ mice. Morphologically, femurs and tibias from C/EBPε^−/−^ mice appeared visibly paler compared with wild-type (C/EBPε^+/+^) controls (Figure 6A). Peripheral blood analysis revealed significant reductions in white blood cells, neutrophils, and T cells. In contrast, monocytes were concomitantly increased (Figure 6B), indicating that C/EBPε regulates multiple hematopoietic lineages.

In CIN mice, the restorative effect of BSP on neutrophil counts was markedly attenuated in C/EBPε^−/−^ mice compared with C/EBPε^+/+^ controls on day 7 post-treatment. Interestingly, BSP treatment increased monocyte numbers in knockout mice, while total WBC and T-cell count remained unchanged. Analysis of bone marrow HSPCs populations revealed that C/EBPε deficiency did not significantly affect LT-HSC and MPP2 cells, but MPP3/4 numbers were significantly reduced (Figure 6C). Moreover, in C/EBPε^−/−^ mice, BSP could not promote HSPCs expansion, demonstrating that BSP-mediated hematopoietic reconstitution depends on C/EBPε. Consistently, C/EBPε ablation nearly abolished expression of downstream effectors LTF and ORM1, significantly reducing LCN2, and markedly upregulating ELANE. Furthermore, BSP treatment failed to modulate these genes in the absence of C/EBPε (Figure 6D). These results establish C/EBPε as a critical mediator of BSP-driven hematopoietic reconstitution and neutrophil regeneration in vivo.

## 4. Discussion

Chemotherapy-induced neutropenia (CIN) is characterized by a severe decrease in circulating neutrophils, significantly increasing the risk of life-threatening infections as neutrophils are the primary responders to infection in the innate immune system [32,33]. G-CSF, a lineage-specific hematopoietic growth factor that primarily acts on committed neutrophil progenitors and later stages to accelerate their proliferation and differentiation, remains the primary treatment for CIN. Given the reported hematopoietic activity of various natural polysaccharides, we investigated whether BSP could promote hematopoietic reconstitution, especially neutrophils recovery in a CIN mouse model.

In our study, BSP led to higher white blood cell (WBC) and neutrophil counts than G-CSF by day 7 in a CIN mouse model (Figure 1B). Although G-CSF exhibited an earlier onset of action, it was primarily used in this study as a positive control for pharmaco-dynamic evaluation. Therefore, a comprehensive systematic comparison with G-CSF was not conducted in subsequent experiments investigating the mechanism of action of BSP.

Beyond the increase in neutrophil numbers, the maturity and phagocytic function of newly generated neutrophils are also critical indicators in CIN. Morphological analysis revealed that BSP promoted the regeneration of mature neutrophils with segmented nu-clei, while functional assays confirmed that these cells retained phagocytic activity (Figure 1D–E). Further testing demonstrated that neutrophils from BSP-treated mice effectively ingested bacteria and conferred protection against LPS challenge—a model of Gram-negative bacterial sepsis (Figure 1G).

Multipotential hematopoietic stem cells sustain lifelong blood cell production. At the cellular level, HSC undergo both asymmetric and symmetric divisions to balance self-renewal and differentiation into diverse hematopoietic lineages [34,35,36,37,38]. As anticipated, BSP treatment stimulated the expansion of HSPCs, as indicated by enhanced proliferation of LSK cells and increased HSPCs numbers (Figure 2).

Furthermore, the reparative function of HSPCs in the hematopoietic system is more critically reflected in their ability to reconstitute blood cells. To validate this, we performed both in vitro CFU assays and in vivo competitive transplantation experiments. CFU analysis further demonstrated that BSP increased GM- and G-type colony formation, suggesting a regenerative effect on granulocyte/macrophage progenitors contributing to neutrophil recovery (Figure 3A). Competitive bone marrow transplantation assays, the gold standard for evaluating hematopoietic reconstitution [22], confirmed that BSP enhances this function of HSPCs after CY-induced bone marrow injury. BSP improved chimerism and corrected impaired myeloid differentiation in recipient mice (Figure 3C,F), highlighting its potential to restore functional hematopoiesis. Typically, 21-day observations are used to assess bone marrow function under steady-state conditions, minimizing acute stress effects. Thus, our data represent a stable hematopoietic state following BSP treatment without depletion of the hematopoietic stem cell pool at this time point.

Next, we employed a PCR array to investigate the potential regulatory mechanisms of BSP. Analysis of HSPCs identified 21 regulated genes, which included surface markers, cytokines (IL-10, IL-1ɑ), C/EBPε, and components of the Notch signaling pathway (Figure 4A). Notch signaling inhibition is known to induce transient expansion of myeloid pro-genitors [39], and our previous work demonstrated that BSP can modulate Notch signaling to promote trained immunity [40]. Among these altered genes, upregulation of the transcription factor C/EBPε during LT-HSCs myelopoiesis has been demonstrated [23,24]. This factor is a member of the C/EBP family, whose various members are known to play vital roles in hematopoiesis and myeloid differentiation [41,42,43,44]. Furthermore, C/EBPε cooperates with transcription factors such as GATA1 and PU.1 to regulate hematopoietic differentiation. Mutations in the bZIP domain of C/EBPε disrupt these interactions, im-pairing granulocyte maturation [45,46]. Gain-of-function mutations in C/EBPε have also been linked to non-canonical inflammasome activation (NLRP3, caspase-5), connecting myeloid differentiation with inflammatory signaling [47]. Additionally, C/EBPε deficiency reduces lipid uptake in macrophages, implicating it in metabolic regulation [48]. Given these multifaceted roles, BSP-induced modulation of C/EBPε may influence both hematopoietic and immune–metabolic pathways.

We further confirmed that BSP upregulates C/EBPε expression in lineage-negative cells. Consistent with this, BSP was found to enhance the expression of its downstream targets LTF and LCN2, while suppressing ELANE (Figure 4B). LTF and LCN2 are known regulators of iron metabolism [49,50], a process crucial for maintaining HSCs quiescence and activation [51]. Multiple serine proteases, including ELANE, collectively cleave and modify CXCL12 variants, significantly impairing the function of the CXCR4/CXCL12 bio-logical axis and leading to abnormal mobilization of hematopoietic stem/progenitor cells [52,53].

Functional validation using shRNA-mediated C/EBPε knockdown demonstrated that BSP’s ability to enhance hematopoietic reconstitution is C/EBPε dependent (Figure 5). Consistently, C/EBPε^−/−^ mice exhibited impaired HSPCs repopulation and neutrophil re-generation, and BSP treatment failed to restore hematopoiesis (Figure 6B-6C). Interestingly, our study revealed that in C/EBPε^−/−^ mice, myeloid differentiation was exclusively skewed toward monocytes. This observation raises important questions regarding the potential role of C/EBPε in monocyte differentiation and function, warranting further investigation in future studies. As expected, the deletion of C/EBPε abolished the expression of LTF and ORM1, reduced LCN2, and markedly upregulated ELANE-a gene highly, specifically expressed in promyelocytes (Figure 6D). The result indicates a maturation arrest at the promyelocyte stage, which is characteristic of certain forms of acute myeloid leukemia (AML). These data collectively identify C/EBPε dysfunction as a pivotal factor in the development of subtypes of AML. Furthermore, BSP holds promise as a therapeutic agent for AML, which merits further investigation.

Previous research has shown that acetylation of C/EBPε in CD34^+^ hematopoietic stem cells promotes terminal neutrophil differentiation, a process modulated by upregulation of SIRT1 and inhibition of p300, both of which suppress C/EBPε acetylation [26,54]. In addition, all-trans retinoic acid (ATRA) has been reported to directly induce C/EBPε expression via the retinoic acid receptor (RAR) signaling pathway [55]. The hematopoietic enhancement induced by polysaccharides is a complex physiological process, and the precise signaling mechanisms remain largely undefined. To our knowledge, no previous studies have described the upstream regulatory pathways of C/EBPε influenced by BSP. Although this study did not directly investigate the mechanism underlying BSP-mediated upregulation of C/EBPε, this represents an important direction for our future work.

**Figure 7:**
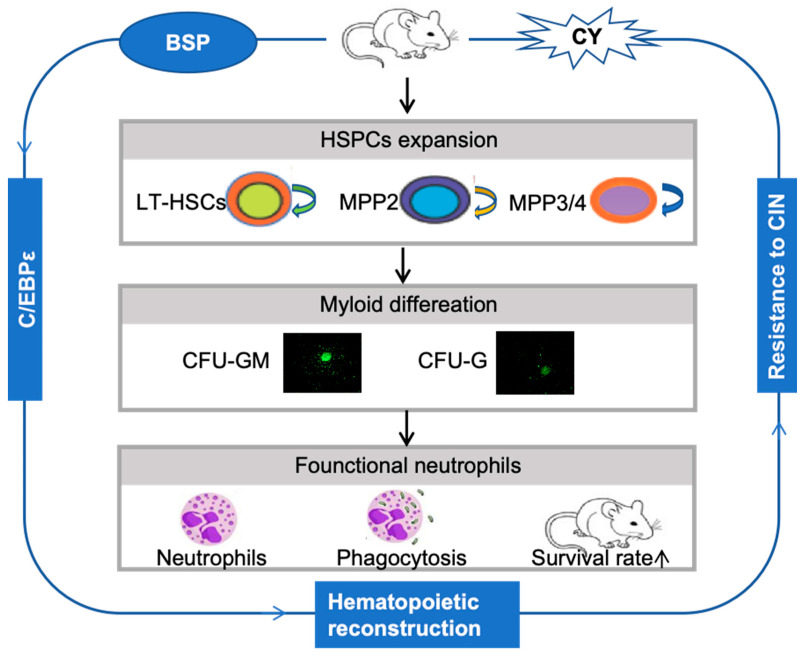
The schematic diagram illustrates that BSP enhances hematopoietic reconstitution of HSPCs and promotes their myeloid differentiation into functional neutrophils by regulating C/EBPε, a process critical for treating neutropenia.

Our study demonstrates that BSP accelerates hematopoietic restoration and generates functional neutrophils capable of protecting against endotoxin-induced mouse death. Mechanistically, BSP promoted HSPCs expansion and increased the number of multipotent progenitors. CFU assays and competitive bone marrow transplantation experiments confirmed that BSP enhances HSPCs reconstitution, an effect associated with upregulation of the transcription factor C/EBPε (Figure 7).

Despite BSP’s strong potential for restoring hematopoiesis through C/EBPε regula-tion with no signs of morbidity in this initial study, a comprehensive toxicological evaluation remains critical for its preclinical development. Notably, polysaccharide drugs face challenges in solubility, stability, delivery efficiency, and batch-to-batch consistency due to their complex physicochemical nature. These limitations underscore the need for optimized formulation strategies in future preclinical studies.

## 5. Conclusions

Collectively, these findings reveal a novel mechanism by which BSP promotes HSPCs expansion and myeloid differentiation through C/EBPε, resulting in functional neutrophil recovery and enhanced host defense in CIN. This work provides a strong rationale for the potential therapeutic application of BSP in chemotherapy-induced neutropenia.

## Data Availability

The original contributions presented in this study are included in the article/Supplementary Material. Further inquiries can be directed to the corresponding author.

## Supplementary Materials

The following supporting information can be downloaded at https://www.mdpi.com/article/10.3390/cells14231888/s1; Figure S1: Extraction process and chemical structure of Bletilla striata polysaccharide; Figure S2: Analysis of peripheral blood at different time points after treatment; Figure S3: Representative cytometer plots for pHrodo FITC-labeled S. aureus at 37 degrees or 4 degrees; Figure S4: The number of clones generated by different doses of BSP; Figure S5: Validation of transduction efficiency by GFP fluorescence and knockdown efficiency by qPCR at 72 h post-shRNA transduction; Table S1: Primers for real-time PCR, C/EBPε shRNA, and gRNA target sequences; Table S2: Gene table of the 37 genes assayed with mouse hematopoiesis RT2 Profiler PCR array. Video S1: LSK cells live image of control mice; Video S2: LSK cells in live image of CY-mice; Video S3: LSK cells in live image of BSP-mice.

## References

[1] Crawford J., Dale D.C., Lyman G.H. (2004). Chemotherapy-Induced Neutropenia: Risks, Consequences, and New Directions for Its Management. Cancer.

[2] Graf T., Enver T. (2009). Forcing Cells to Change Lineages. Nature.

[3] Koinis F., Nintos G., Georgoulias V., Kotsakis A. (2015). Therapeutic Strategies for Chemotherapy-Induced Neutropenia in Patients with Solid Tumors. Expert Opin. Pharmacother..

[4] Yu V.W.C., Scadden D.T. (2016). Hematopoietic Stem Cell and Its Bone Marrow Niche. Curr. Top. Dev. Biol..

[5] Seita J., Weissman I.L. (2010). Hematopoietic Stem Cell: Self-Renewal versus Differentiation. Wiley Interdiscip. Rev. Syst. Biol. Med..

[6] Höfer T., Rodewald H.-R. (2018). Differentiation-Based Model of Hematopoietic Stem Cell Functions and Lineage Pathways. Blood.

[7] Kawamoto H., Ikawa T., Masuda K., Wada H., Katsura Y. (2010). A Map for Lineage Restriction of Progenitors during Hematopoiesis: The Essence of the Myeloid-Based Model. Immunol. Rev..

[8] Theilgaard-Mönch K., Jacobsen L.C., Borup R., Rasmussen T., Bjerregaard M.D., Nielsen F.C., Cowland J.B., Borregaard N. (2005). The Transcriptional Program of Terminal Granulocytic Differentiation. Blood.

[9] Panopoulos A.D., Watowich S.S. (2008). Granulocyte Colony-Stimulating Factor: Molecular Mechanisms of Action during Steady State and “emergency” Hematopoiesis. Cytokine.

[10] Abdel-Azim H., Sun W., Wu L. (2019). Strategies to Generate Functionally Normal Neutrophils to Reduce Infection and Infection-Related Mortality in Cancer Chemotherapy. Pharmacol. Ther..

[11] Dick E.P., Prince L.R., Sabroe I. (2008). Ex Vivo-Expanded Bone Marrow CD34+ Derived Neutrophils Have Limited Bactericidal Ability. Stem Cells.

[12] Cardot-Ruffino V., Bollenrucher N., Delius L., Wang S.J., Brais L.K., Remland J., Keheler C.E., Sullivan K.M., Abrams T.A., Biller L.H. (2023). G-CSF Rescue of FOLFIRINOX-Induced Neutropenia Leads to Systemic Immune Suppression in Mice and Humans. J. Immunother. Cancer.

[13] Kuśnierek M., Czerwińska M. (2024). Lycium barbarum polysaccharide fraction–isolation from fruits and impact on the secretion of inflammatory mediators by human mononuclear cells and neutrophils. Prospect. Pharm. Sci..

[14] Czerwińska M., Kuśnierek M. (2024). Owoce Lycium barbarum—skład chemiczny i aktywność jagód goji—od tradycji do badań klinicznych. Prospect. Pharm. Sci..

[15] Wang C., Sun J., Luo Y., Xue W., Diao H., Dong L., Chen J., Zhang J. (2006). A Polysaccharide Isolated from the Medicinal Herb Bletilla Striata Induces Endothelial Cells Proliferation and Vascular Endothelial Growth Factor Expression in Vitro. Biotechnol. Lett..

[16] Wu X., Xin M., Chen H., Yang L., Jiang H. (2010). Novel Mucoadhesive Polysaccharide Isolated from Bletilla Striata Improves the Intraocular Penetration and Efficacy of Levofloxacin in the Topical Treatment of Experimental Bacterial Keratitis. J. Pharm. Pharmacol..

[17] Wang Y., Han S., Li R., Cui B., Ma X., Qi X., Hou Q., Lin M., Bai J., Li S. (2019). Structural Characterization and Immunological Activity of Polysaccharides from the Tuber of Bletilla Striata. Int. J. Biol. Macromol..

[18] Fang H., Xie X., Liu P., Rao Y., Cui Y., Yang S., Yu J., Luo Y., Feng Y. (2020). Ziyuglycoside II Alleviates Cyclophosphamide-Induced Leukopenia in Mice via Regulation of HSPCs Proliferation and Differentiation. Biomed. Pharmacother..

[19] Ding W., Shimada H., Li L., Mittal R., Zhang X., Shudo K., He Q., Prasadarao N.V., Wu L. (2013). Retinoid Agonist Am80-Enhanced Neutrophil Bactericidal Activity Arising from Granulopoiesis in Vitro and in a Neutropenic Mouse Model. Blood.

[20] Villarino N., Brown S.A., Martín-Jiménez T. (2012). Pharmacokinetics of Tulathromycin in Healthy and Neutropenic Mice Challenged Intranasally with Lipopolysaccharide from Escherichia Coli. Antimicrob. Agents Chemother..

[21] Moynihan J., Cohen N. (1989). The Kinetics of Recovery of Leukocyte Number and Lymphocyte Function Following an Injection of a Single High Dose of Cyclophosphamide in C3H/HeJ Mice. Int. J. Immunopharmacol..

[22] Hétu-Arbour R., Bouali S., Heinonen K.M. (2021). Experimental Competitive Bone Marrow Transplant Assays. Methods Mol. Biol..

[23] de Roo J.J.D., Staal F.J.T. (2020). Cell Signaling Pathway Reporters in Adult Hematopoietic Stem Cells. Cells.

[24] Mitrou Lis I., Ruppova K., Wang B., Chen L.-S., Grzybek M., Grinenko T., Eugster A., Troullinaki M., Palladini A., Kourtzelis I. (2018). Modulation of Myelopoiesis Progenitors Is an Integral Component of Trained Immunity. Cell.

[25] Gery S., Park D.J., Vuong P.T., Virk R.K., Muller C.I., Hofmann W.-K., Koeffler H.P. (2007). RTP801 Is a Novel Retinoic Acid-Responsive Gene Associated with Myeloid Differentiation. Exp. Hematol..

[26] Bartels M., Govers A.M., Fleskens V., Lourenço A.R., Pals C.E., Vervoort S.J., van Gent R., Brenkman A.B., Bierings M.B., Ackerman S.J. (2015). Acetylation of C/EBPε Is a Prerequisite for Terminal Neutrophil Differentiation. Blood.

[27] Akagi T., Thoennissen N.H., George A., Crooks G., Song J.H., Okamoto R., Nowak D., Gombart A.F., Koeffler H.P. (2010). In Vivo Deficiency of Both C/EBPβ and C/EBPε Results in Highly Defective Myeloid Differentiation and Lack of Cytokine Response. PLoS ONE.

[28] Chumakov A.M., Grillier I., Chumakova E., Chih D., Slater J., Koeffler H.P. (1997). Cloning of the Novel Human Myeloid-Cell-Specific C/EBP-Epsilon Transcription Factor. Mol. Cell. Biol..

[29] Lekstrom-Himes J.A., Dorman S.E., Kopar P., Holland S.M., Gallin J.I. (1999). Neutrophil-Specific Granule Deficiency Results from a Novel Mutation with Loss of Function of the Transcription Factor CCAAT/Enhancer Binding Protein Epsilon. J. Exp. Med..

[30] Verbeek W., Lekstrom-Himes J., Park D.J., Dang P.M., Vuong P.T., Kawano S., Babior B.M., Xanthopoulos K., Koeffler H.P. (1999). Myeloid Transcription Factor C/EBPepsilon Is Involved in the Positive Regulation of Lactoferrin Gene Expression in Neutrophils. Blood.

[31] Yamanaka R., Barlow C., Lekstrom-Himes J., Castilla L.H., Liu P.P., Eckhaus M., Decker T., Wynshaw-Boris A., Xanthopoulos K.G. (1997). Impaired Granulopoiesis, Myelodysplasia, and Early Lethality in CCAAT/Enhancer Binding Protein Epsilon-Deficient Mice. Proc. Natl. Acad. Sci. USA.

[32] Bardoel B.W., Kenny E.F., Sollberger G., Zychlinsky A. (2014). The Balancing Act of Neutrophils. Cell Host Microbe.

[33] Wright H.L., Moots R.J., Bucknall R.C., Edwards S.W. (2010). Neutrophil function in inflammation and inflammatory diseases. Rheumatology.

[34] Busch K., Klapproth K., Barile M., Flossdorf M., Holland-Letz T., Schlenner S.M., Reth M., Höfer T., Rodewald H.-R. (2015). Fundamental Properties of Unperturbed Haematopoiesis from Stem Cells in Vivo. Nature.

[35] Morrison S.J., Kimble J. (2006). Asymmetric and Symmetric Stem-Cell Divisions in Development and Cancer. Nature.

[36] Tajbakhsh S., Rocheteau P., Le Roux I. (2009). Asymmetric Cell Divisions and Asymmetric Cell Fates. Annu. Rev. Cell Dev. Biol..

[37] Doulatov S., Notta F., Laurenti E., Dick J.E. (2012). Hematopoiesis: A Human Perspective. Cell Stem Cell.

[38] Akashi K., Traver D., Miyamoto T., Weissman I.L. (2000). A Clonogenic Common Myeloid Progenitor That Gives Rise to All Myeloid Lineages. Nature.

[39] Wang W., Yu S., Myers J., Wang Y., Xin W.W., Albakri M., Xin A.W., Li M., Huang A.Y., Xin W. (2017). Notch2 Blockade Enhances Hematopoietic Stem Cell Mobilization and Homing. Haematologica.

[40] Zhang J., Zhong C., Chen L., Luo Y., Tang L., Yang J., Jia J., Xie X., Liu P., Yu J. (2025). Bletilla Striata Polysaccharide-Mediated Trained Immunity Drives the Hematopoietic Progenitors’ Expansion and Myelopoiesis. Int. Immunopharmacol..

[41] Singh A.K., Thacker G., Upadhyay V., Mishra M., Sharma A., Sethi A., Chowdhury S., Siddiqui S., Verma S.P., Pandey A. (2024). Nemo-like Kinase Blocks Myeloid Differentiation by Targeting Tumor Suppressor C/EBPα in AML. FEBS J..

[42] Mildner A., Schönheit J., Giladi A., David E., Lara-Astiaso D., Lorenzo-Vivas E., Paul F., Chappell-Maor L., Priller J., Leutz A. (2017). Genomic Characterization of Murine Monocytes Reveals C/EBPβ Transcription Factor Dependence of Ly6C-Cells. Immunity.

[43] Suh H.C., Gooya J., Renn K., Friedman A.D., Johnson P.F., Keller J.R. (2006). C/EBPalpha Determines Hematopoietic Cell Fate in Multipotential Progenitor Cells by Inhibiting Erythroid Differentiation and Inducing Myeloid Differentiation. Blood.

[44] Hayashi Y., Hirai H., Kamio N., Yao H., Yoshioka S., Miura Y., Ashihara E., Fujiyama Y., Tenen D.G., Maekawa T. (2013). C/EBPβ Promotes BCR-ABL-Mediated Myeloid Expansion and Leukemic Stem Cell Exhaustion. Leukemia.

[45] Wada T., Akagi T. (2016). Role of the Leucine Zipper Domain of CCAAT/Enhancer Binding Protein-Epsilon (C/EBPε) in Neutrophil-Specific Granule Deficiency. Crit. Rev. Immunol..

[46] Tamaru T., Katayama R., Momokino J., Maruoka Y., Ueda A., Kanegane H., Wada T., Bukhari S.T.A., Banday A.Z., Akagi T. (2025). Genotype-Phenotype Correlations in Specific Granule Deficiency: Loss of DNA-Binding Ability and Impaired Nuclear Localization Cause Severe Manifestations Due to the c.655_665del CEBPE Variant. Clin. Exp. Immunol..

[47] Göös H., Fogarty C.L., Sahu B., Plagnol V., Rajamäki K., Nurmi K., Liu X., Einarsdottir E., Jouppila A., Pettersson T. (2019). Gain-of-Function CEBPE Mutation Causes Noncanonical Autoinflammatory Inflammasomopathy. J. Allergy Clin. Immunol..

[48] Okamoto R., Gery S., Gombart A.F., Wang X., Castellani L.W., Akagi T., Chen S., Arditi M., Ho Q., Lusis A.J. (2014). Deficiency of CCAAT/Enhancer Binding Protein-Epsilon Reduces Atherosclerotic Lesions in LDLR-/- Mice. PLoS ONE.

[49] Karbakhsh Ravari F., Ghasemi Gorji M., Rafiei A. (2025). From Iron-Driven Cell Death to Clot Formation: The Emerging Role of Ferroptosis in Thrombogenesis. Biomed. Pharmacother..

[50] Valenti P., Antonini G. (2005). Lactoferrin: An Important Host Defence against Microbial and Viral Attack. Cell. Mol. Life Sci..

[51] Yi W., Zhang J., Huang Y., Zhan Q., Zou M., Cheng X., Zhang X., Yin Z., Tao S., Cheng H. (2024). Ferritin-mediated mitochondrial iron homeostasis is essential for the survival of hematopoietic stem cells and leukemic stem cells. Leukemia.

[52] Nie Y., Han Y.-C., Zou Y.-R. (2008). CXCR4 is required for the quiescence of primitive hematopoietic cells. J. Exp. Med..

[53] Cho S.Y., Xu M., Roboz J., Lu M., Mascarenhas J., Hoffman R. (2010). The Effect of CXCL12 Processing on CD34+ Cell Migration in Myeloproliferative Neoplasms. Cancer Res..

[54] Cheng S., Zhu J., Bian Y., Yao J., Zhang W., Yin S., Kuang T., Xian L., Liang H. (2025). C/EBPε and Its Acetylation in PMN Enhance the Tolerance to Trauma. Clin. Exp. Immunol..

[55] Cai R., Cai X., Chen B., Xu W., Lu J. (2010). C/EBPε Participates in All-Trans Retinoic Acid Induction of PI3Kγ in U937 Cells via an Intronic Matrix Attachment Region Sequence. Mol. Biol. Rep..

