# Supplementary material for: Bletilla Striata Polysaccharide Alleviates Neutropenia by Promoting C/EBPε-Dependent Hematopoietic Reconstitution
Source: Cells. 2025 Nov 28;14(23):1888. doi: 10.3390/cells14231888 (PMC12691188; doi:10.3390/cells14231888)
Supplement: Supplementary file 1 [file cells-14-01888-s001.zip › cells-3915197-supplementary.pdf]

Bletilla Striata Polysaccharide Alleviates Neutropenia by Promoting C/EBPε-Dependent Hematopoietic Reconstitution

Yaru Cui 1,2,3, Yingying Luo 1,2,3, Cheng Zhang 3, Dan Shan 3, Yulin Feng 1, Shilin Yang 1, Lanying Chen 1,2,* and Jun Yu 3,*

1 National Pharmaceutical Engineering Center for Solid Preparation in Chinese Herbal Medicine, Jiangxi University of Chinese Medicine, Nanchang 330006, China; cuiyaru@jxutcm.edu.cn (Y.C.)

2 Jiangxi Provincial Key Laboratory of Effective Material Basis of TCM, Jiangxi University of Chinese Medicine, Nanchang 330004, China.

3 Department of Cardiovascular Sciences and Center for Metabolic Disease Research, Lewis Katz School of Medicine, Temple University, Philadelphia, PA 19140, USA

*** Correspondence:** 19960228@jxutcm.edu.cn (L.C.); jun.yu@temple.edu (J.Y.)

Supplementary Figures

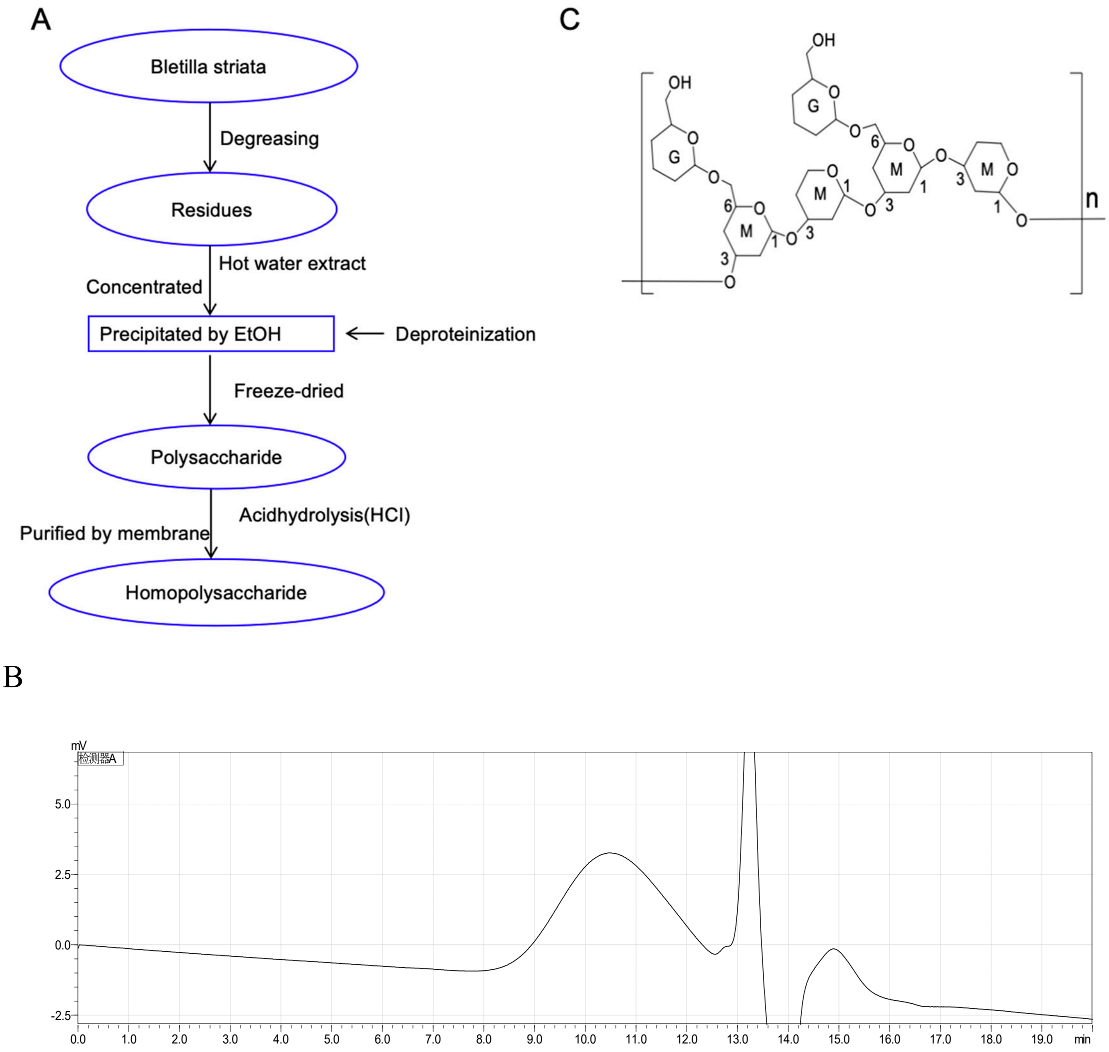

Figure S1. Extraction process and chemical structure of Bletilla striata polysaccharide.

(A) The process of isolating and purifying homopolysaccharides from Bletilla striata.

(B) The HPGPC of Bletilla striata polysaccharide.

(C) Chemical structure of Bletilla striata polysaccharide.

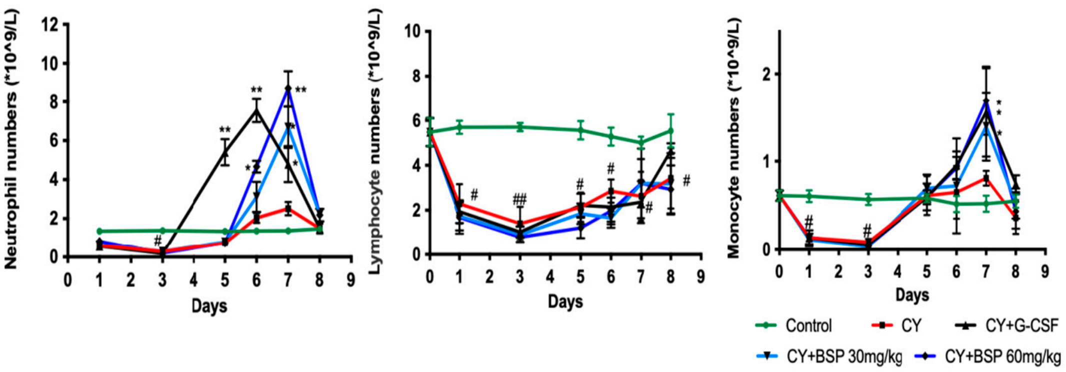

Figure S2. Analysis of peripheral blood at different time points. Related to Figure 1B. Representative peripheral blood neutrophil, lymphocyte and monocyte analysis at different time points. Data are expressed as the mean ± SD, *n*=6; Statistical significance was determined by one-way ANOVA with the Bonferroni's post hoc test; # $P < 0.05$ or ## $P < 0.01$ versus control group. * $P < 0.05$ or ** $P < 0.01$ versus CY group.

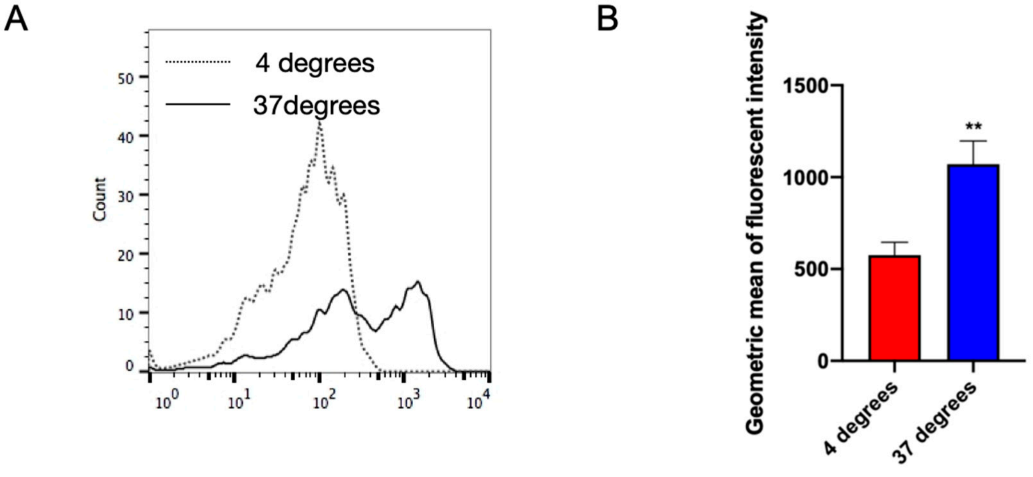

Figure S3. Representative cytometer plots for pHrodo FITC-labeled S. aureus at 37 degrees or 4 degrees. Data are expressed as the mean ± SD, n=3; Statistical significance was determined by unpaired Student's t-test with the Bonferroni's post hoc test; ** $P < 0.01$ versus 4 degrees. Related to Figure 1E.

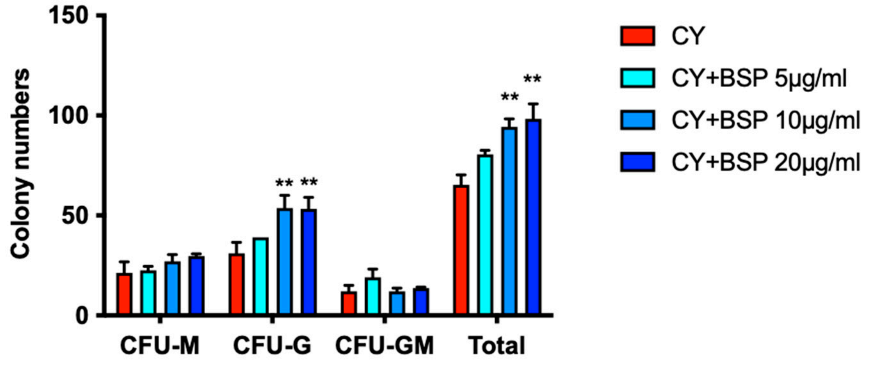

Figure S4. The number of clones generated by different doses of BSP. Related to Figure 3A. Sorted LSK cells were treated with different BSP doses and then plated for the CFU assay. Counted the colony number at 8 days and decided to choose BSP 20 μg/ml dose for the next assay. Data is expressed as the mean ± SD, n=3. Statistical significance was determined by one-way ANOVA with the Bonferroni's post hoc test; ** $P < 0.01$ versus CY group.

A

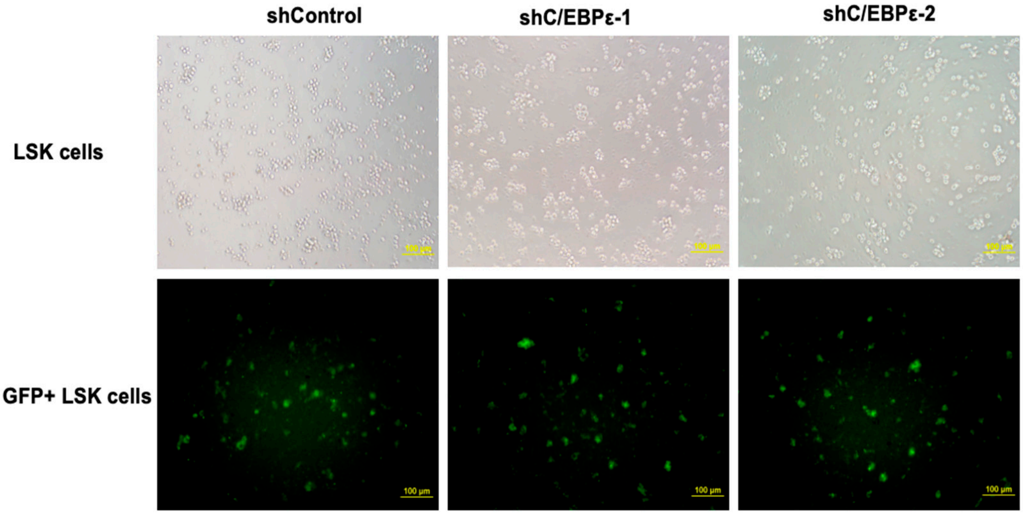

B

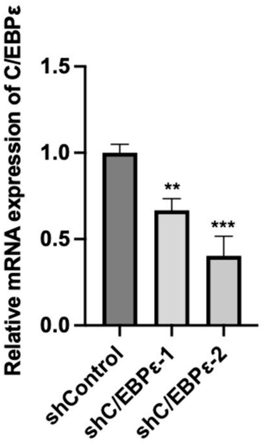

Figure S5. (A) GFP expression in LSK cells after infection 72 h. (B)The effect of the shRNA on C/EBPε mRNA in LSK cells after infection 72 h. Data is expressed as the mean ± SD, *n*=3. Statistical significance was determined by one-way ANOVA with the Bonferroni's post hoc test; ** $P < 0.01$ or *** $P < 0.001$ versus shControl. Related to Figure 5.

Table S1. Primers for Real-time PCR and C/EBPε shRNA and gRNA target sequences.

Genes	Forward 5'-3'	Reverse 5'-3'
β--actin	ACCTTCTACAATGAGCTGCG	CTGGATGGCTACGTACATGG
LTF	TGAGGCCCTTGGACTCTGT	ACCCACTTTTCTCATCTCGTTC
ITGAM	ATGGACGCTGATGGCAATACC	TCCCCATTCACGTCTCCCA
LYZ1	GAGACCGAAGCACCGACTATG	CGGTTTTGACATTGTGTTCGC
NCF1	ACACCTTCATTCGCCATATTGC	TCGGTGAATTTTCTGTAGACCAC
ORM1	CGAGTACAGGCAGGCAATTCA	ACCTATTGTTTGAGACTCCCGA
LCN2	TGGCCCTGAGTGTCATGTG	CTCTTGTAGCTCATAGATGGTGC
ELANE	CCTTGGCAGACTATCCAGCC	GACATGACGAAGTTCCTGGCA
C/EBPε	GTGAACAAAGATAGCCTGGAGTACCG	GACCTTCTGCTGAGTCTCCATAATGC
C/EBPε-shRNA1	GAGGGCAAACCTAGGGCAAATCT	
C/EBPε-shRNA2	GAAGGCAGTGAACAAAGAT	
C/EBPε gRNA-B1	CTCCGTCACCAACTCCTACGTGG	
C/EBPε gRNA-B2	AGCACACACTCGATGTACAAAGG	

Table S2. Gene table of the 37 genes assayed with mouse hematopoiesis RT2 Profiler PCR array. Related to Figure 4.

Gene	Description	Fold change	P -value
Il1a	Interleukin 1 alpha	12.7286	0.063837
Cd14	CD14 antigen	8.4172	0.124646
Notch4	Notch gene homolog 4 (Drosophila)	2.8879	0.318265
Kitl	Kit ligand	2.4005	0.557708
Cebpe	CCAAT/enhancer binding protein (C/EBP), epsilon	2.3349	0.297153
Il10	Interleukin 10	2.2038	0.08661
Kdr	Kinase insert domain protein receptor	2.1189	0.354568
Ccr1	Chemokine (C-C motif) receptor 1	2.061	0.281598
Cd2	CD2 antigen	1.9862	0.310976
Cd80	CD80 antigen	1.6586	0.123801
Cd3g	CD3 antigen, gamma polypeptide	1.6021	0.587283
Cd4	CD4 antigen	1.5984	0.409242
Nos2	Nitric oxide synthase 2, inducible	1.3755	0.466481
Cbfb	Core binding factor beta	1.3566	0.086091
Blnk	B-cell linker	1.3287	0.432014
Mmp9	Matrix metallopeptidase 9	1.3226	0.160517
Stim2	Stromal interaction molecule 2	1.3104	0.120615
Fzd1	Frizzled homolog 1 (Drosophila)	1.3013	0.68717
Cd164	CD164 antigen	1.257	0.108226
Lrmp	Lymphoid-restricted membrane protein	1.2454	0.191862
Flt3l	FMS-like tyrosine kinase 3 ligand	1.2114	0.339717
Sfxn1	Sideroflexin 1	1.1173	0.017712
Dll1	Delta-like 1 (Drosophila)	-7.3276	0.002433
Trim10	Tripartite motif-containing 10	-6.4086	0.036552
Cd27	CD27 antigen	-3.9816	0.376186
Tnfsf11	Tumor necrosis factor (ligand) superfamily, member 11	-1.7695	0.058737
Inhba	Inhibin alpha	-1.6857	0.054525

Il11	Interleukin 11	-1.6283	0.834748
Mal	Myelin and lymphocyte protein, T-cell differentiation protein	-1.5511	0.0319
Tek	Endothelial-specific receptor tyrosine kinase	-1.4439	0.202145
Jag2	Jagged 2	-1.3348	0.383275
Cd86	CD86 antigen	-1.3104	0.543061
Angpt1	Angiopoietin 1	-1.2716	0.319383
Pax5	Paired box gene 5	-1.2541	0.735184
Socs5	Suppressor of cytokine signaling 5	-1.2454	0.10215
Notch2	Notch gene homolog 2 (Drosophila)	-1.217	0.015768
Hdac4	Histone deacetylase 4	-1.2002	0.196538

Supplementary Video Legends:

Video S1. The representative time-lapse movie tracks the division of LSK+ cells sorted from the BM of control mice ex vivo over 48 h in culture.

Video S2. The representative time-lapse movie tracks the division of LSK+ cells sorted from the BM of CY-treated mice ex vivo over 48 h in culture.

Video S3. The representative time-lapse movie tracks the division of LSK+ cells sorted from the BM of BSP-treated mice ex vivo over 48 h in culture.
